# High-dose chemotherapy followed by autologous stem cell transplantation for relapsed/refractory primary mediastinal large B-cell lymphoma

**DOI:** 10.1038/bcj.2015.101

**Published:** 2015-12-04

**Authors:** T Aoki, K Shimada, R Suzuki, K Izutsu, A Tomita, Y Maeda, J Takizawa, K Mitani, T Igarashi, K Sakai, K Miyazaki, K Mihara, K Ohmachi, N Nakamura, H Takasaki, H Kiyoi, S Nakamura, T Kinoshita, M Ogura

**Affiliations:** 1 Department of Hematology and Oncology, Nagoya Daini Red Cross Hospital, Nagoya, Japan; 2 Department of Hematology and Oncology, Nagoya University Graduate School of Medicine, Nagoya, Japan; 3 Cancer Center, Shimane University Hospital, Izumo, Japan; 4 Department of Hematology, Toranomon Hospital, Tokyo, Japan; 5 Department of Hematology and Oncology, Okayama University Hospital, Okayama, Japan; 6 Department of Hematology, Endocrinology and Metabolism, Niigata University Faculty of Medicine, Niigata, Japan; 7 Department of Hematology and Oncology, Dokkyo Medical University School of Medicine, Tochigi, Japan; 8 Department of Hematology and Oncology, Gunma Cancer Center, Oota, Japan; 9 Department of Biomedical Laboratory Sciences, Shinshu University School of Medicine, Matsumoto, Japan; 10 Department of Hematology, Shinshu University School of Medicine, Matsumoto, Japan; 11 Department of Hematology and Oncology, Mie University Graduate School of Medicine, Tsu, Japan; 12 Department of Hematology, Hiroshima University Hospital, Hiroshima, Japan; 13 Department of Hematology, Tokai University, Isehara, Japan; 14 Department of Pathology, Tokai University, Isehara, Japan; 15 Department of Medical Oncology, Kanagawa Cancer Center, Yokohama, Japan; 16 Department of Pathology and Clinical Laboratories, Nagoya University Hospital, Nagoya, Japan; 17 Department of Hematology and Cell Therapy, Aichi Cancer Center, Nagoya, Japan; 18 Department of Hematology, Tokai Central Hospital, Kakamigahara, Japan

As a different entity of diffuse large B-cell lymphoma (DLBCL) according to the current World Health Organization classification,^1^ primary mediastinal large B-cell lymphoma (PMBL) is different from DLBCL in terms of clinical, immunohistochemical and genetic features.^2^ Despite a remarkable advance with first-line treatment of PMBL patients in the rituximab era,^3, 4, 5, 6, 7^ 10–30% of patients still experience progression or relapse. Although patients with relapsed or refractory PMBL are often treated with high-dose therapy followed by autologous stem cell transplantation (HDT/ASCT) after salvage treatment, the progression-free survival (PFS) at 5 years of 27% is unsatisfactory compared with DLBCL in the pre-rituximab era.^8, 9, 10^ Moreover, information regarding outcomes after HDT/ASCT for relapsed or refractory PMBL is limited in the rituximab era. Therefore, clarifying the current role of HDT/ASCT is vital to establish the optimal treatment strategy.

Recently, we published the results of a multicenter retrospective study for newly diagnosed PMBL patients in Japan and described the outcome following first-line treatment.^6^ This report describes subgroup analyses of our recent retrospective study, focusing on the primary end point for patients treated with HDT/ASCT after relapse or refractory disease to clarify the clinical outcomes and the role of HDT/ASCT for PMBL patients with relapsed or refractory disease. Detailed information about the patients, data collection and central pathological review of our analysis were described previously.^6^ Information about treatment and assessment for patients with relapse and refractory disease and statistical method are also described in Supplementary Method 1. The study protocol was approved by the Institutional Review Board of Nagoya Daini Red Cross Hospital (where this study was organized) and each participating hospital based on the Ethical Guideline for Epidemiologic Research from the Ministry of Health, Labor and Welfare in Japan. The study complied with all the provisions of the Declaration of Helsinki.

We identified a total of 44 PMBL patients treated with HDT/ASCT after first relapse or primary refractory disease between 1996 and 2012, and retrospectively analyzed. Patient characteristics are summarized in Table 1. The median time from initial diagnosis to the first relapse or refractory disease was 8 months. Relapse or refractory disease occurred <12 months from initial diagnosis in 66% of patients. The patients with primary refractory disease comprised 41% of the population. The median age at relapse was 26.5 (range, 17–59) years, and female patients were predominant (59%). Stage I/II at relapse was also predominant (60%). Of 44 PMBL patients with relapse or refractory disease, 34 (79%) and 2 (5%) patients had a relapse in the mediastinum or central nervous system, respectively. Twenty-nine patients (66%) had received rituximab-containing chemotherapy as the first-line treatment. Ten patients (23%) had received radiotherapy (RT) as part of the first-line treatment. Eleven patients (25%) had received RT as part of the second-line treatment.

As a salvage regimen, a high-dose (⩾2 g/m^2^) cytarabine-based regimen and an ICE (ifosfamide, etoposide and carboplatin)-based regimen were used in 49% and 19% of patients, respectively. As conditioning regimen, the BEAM (carmustine, etoposide, cytarabine and melphalan)-based protocol was the most frequently used (41%), followed by the MCEC (ranimustine, cyclophosphamide, etoposide and carboplatin)-based regimen^11^ (25%) and the LEED regimen (cyclophosphamide, etoposide, melphalan and dexamethasone)^12^ (20%).

Patient characteristics according to chemo-sensitivity are shown in Table 1. Advanced-stage patients were significantly predominant in chemo-refractory group than the chemo-sensitive group (69% vs 27%, *P*=0.016). No other significant differences were found between the two groups.

The overall response rate after HDT/ASCT was 77.2% (complete remission, 63.6%). With a median follow-up of 53.5 months in surviving patients, the overall survival (OS) and PFS at 4 years were 70% and 61%, respectively (Figures 1a and b). The median OS after relapse or progression was not reached. The OS at 4 years was 73% in relapsed patients and 65% in patients with primary refractory disease (*P*=0.53, Figure 1c). OS did not significantly differ between patients irrespective of rituximab-containing salvage treatment (*P*=0.49, Figure 1d).

A chemo-refractory relapse (*N*=13) was associated with a shorter OS and PFS when compared with chemo-sensitive relapse (*N*=31; 4-year OS: 80% vs 50%, chemo-sensitive vs chemo-refractory, respectively, *P*=0.018, Figure 1e; 4-year PFS: 69% vs 45%, *P*=0.098, Figure 1f). Meanwhile, patients who experienced a late relapse after 12 months from initial treatment (*N*=15) showed excellent outcomes when compared with those who experienced an early relapse (*N*=29) (4-year OS: 60% vs 92%, early relapse vs late relapse, respectively, *P*=0.07; Figure 1g; 4-year PFS: 52% vs 84%, *P*=0.06; Figure 1h). For five patients who underwent allogeneic hematopoietic stem cell transplantation (allo-HSCT) as salvage treatment after HDT/ASCT (*N*=5), about half the patients achieved a durable response with allo-HSCT (Supplementary Figure S1).

Regarding non-relapse mortality after HDT/ASCT, one patient died <100 days as a result of toxicity from transplantation due to infection. Second malignancy developed in one patient after 14 months from HDT/ASCT.

Analysis of prognostic factors for PFS and OS in PMBL patients with relapsed or refractory disease is shown in Supplementary Table S1. Although there was a trend for shorter OS and PFS for patients with early relapse <12 months (*P*=0.097 and 0.097, respectively), no significant prognostic factor was identified in this study. Prior rituximab treatment and RT did not affect the survival of PMBL patients with relapsed or refractory disease in this study.

The present study demonstrated that HDT/ASCT was effective and could be curative after relapse or refractory disease in a substantial number of PMBL patients. In a past study, PMBL patients with relapsed or refractory disease had inferior outcomes compared with DLBCL patients.^8^ However, the OS in PMBL patients treated with HDT/ASCT after relapsed or refractory disease in this study was comparable to that in DLBCL patients with relapsed or refractory disease.^13^ In the absence of randomized trials, a relatively large retrospective study, such as the present study, represents an important source of evidence that can contribute to the establishment of rational treatment recommendations for relapsed or refractory PMBL.

In the present study, there was a trend of shorter OS and PFS for patients who experienced early relapse <12 months after diagnosis although it was not significant probably owing to small number of patients. Notably, >80% of patients treated with HDT/ASCT showed a curative potential when they experienced a late relapse. This result is consistent with findings from the CORAL (Collaborative Trial in Relapsed Aggressive Lymphoma) study of relapsed DLBCL.^13^

Response to salvage chemotherapy is another important issue. The OS at 4 years for patients with chemo-sensitive disease was significantly higher than that of patients with chemo-refractory disease (80% and 50%, respectively) in the present study. These results are consistent with those of a previous study.^10^ Therefore, if stem cell transplantation is considered, outcomes are expected to be best in chemo-sensitive patients at the time of second-line therapy. Novel drugs targeting CD30 or PD-1 have been developed recently,^14^ and an innovative approach including these novel drugs should be explored to increase the response rate of salvage regimens for PMBL patients with relapsed or refractory disease.

Treatment-related toxicities are another important issue to consider when weighing the benefits of HDT/ASCT. In the current study, treatment-related mortality at <100 days was 2.3% (*N*=1) in the HDT/ASCT group, which is consistent with prior reports in DLBCL.^13^ Moreover, only one patient developed a second malignancy in the HDT/ASCT group in the present study. However, longer follow-up is required to evaluate the incidence of late toxicity such as a second malignancy.

The role of allo-HSCT for relapsed and refractory PMBL after HDT/ASCT has not been fully investigated. Nath *et al.*^9^ described the efficacy of allo-HSCT as salvage therapy for a relapsed PMBL patient as a case report. In the present study, about half of the patients who underwent allo-HSCT as salvage treatment after HDT/ASCT achieved durable remission. Therefore, allo-HSCT could be curative, at least in a portion of patients failing HDT/ASCT.

Although the present study provides novel information regarding PMBL, several limitations should be discussed. First, this was a retrospective study with possible unrecognized biases. Second, patients received various regimens of chemotherapy according to each institution's preferred strategy; therefore, treatment outcomes may have been overestimated or underestimated. Finally, we used computed tomography (CT) for response assessment because a large number of PMBL patients in this study were treated before positron emission tomography/CT (PET/CT) became widespread in Japan. PET/CT images may be superior compared with CT for distinguishing the tumor activity of the residual mediastinal mass.^15^ Therefore, the response assessed with CT in this study may have been underestimated for patients with a residual mediastinal mass after treatment.

In conclusion, HDT/ASCT is a good treatment option for relapsed or refractory PMBL patients, especially those who experienced a relapse ⩾12 months after diagnosis. However, considering the poor outcome of chemo-refractory patients and patients who experience an early relapse (<12 months after diagnosis), efforts should be made to improve the response rate to salvage chemotherapy before administering HDT/ASCT. These findings require validation in future prospective studies.

## Figures and Tables

**Figure 1:**
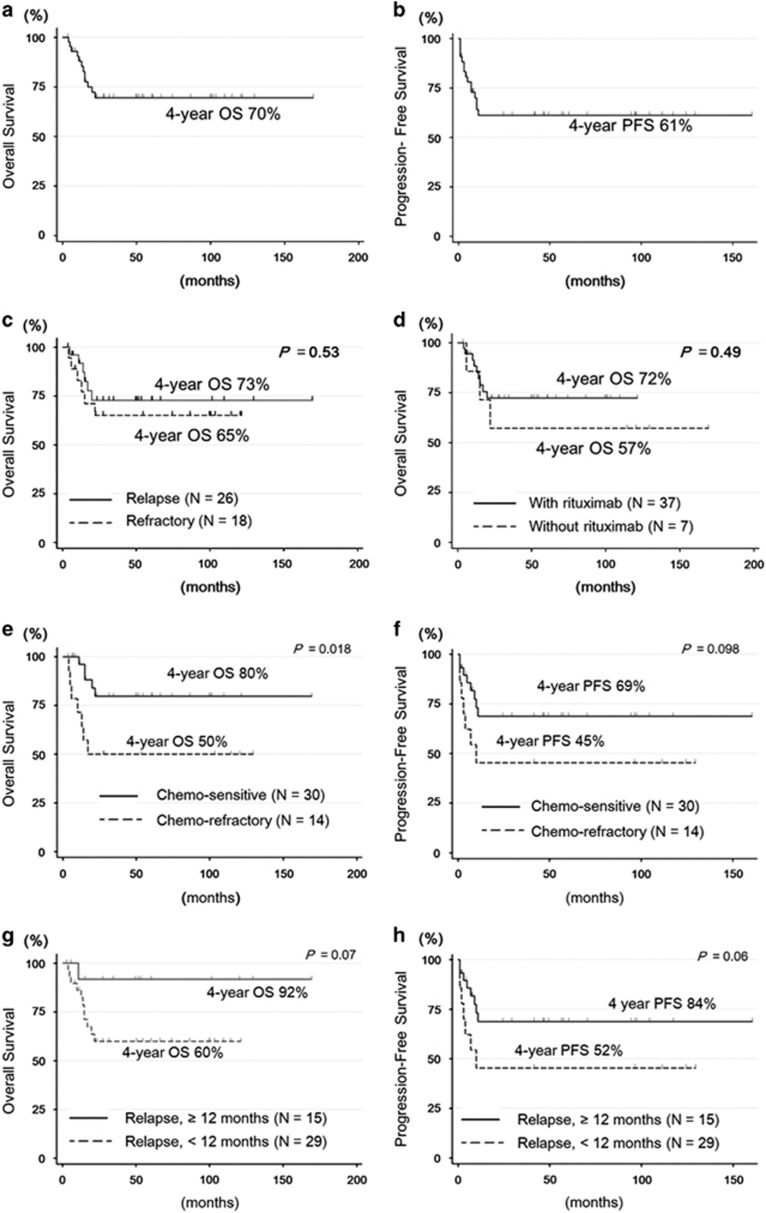
Survival after first relapse or progression in PMBL patients treated with HDT/ASCT. (**a**) OS of all patients, (**b**) PFS of all patients, (**c**) OS according to disease status, (**d**) OS according to rituximab-containing treatment, (**e**) OS according to chemotherapy responsiveness at transplantation, (**f**) PFS according to chemotherapy responsiveness at transplantation, (**g**) OS according to relapse <12 months after diagnosis and (**h**) PFS according to relapse <12 months after diagnosis.

**Table 1:** Patient characteristics

*Characteristics*	*HDT/ASCT*		*Chemo-sensitive*		*Chemo-refractory*		
	*No.*	*%*	*No.*	*%*	*No.*	*%*	P*-value*
No. of patients	44	100	30	68	14	32	

*Age at relapse (years)*							
Median	26.5		29.5		26.5		
Range	17–59		19–59		17–48		
⩾30 years	14	32	10	33	4	12	>0.99

*Sex*							
Male	18	41	12	40	6	43	0.748
Female	26	59	18	60	8	57	

*Stage at relapse*							
I/II	26	60	8	27	9	69	0.016
Relapse at mediastinum	34	79	23	77	11	85	0.699
CNS relapse	2	5	2	6.6	0	0	>0.99

*PS at diagnosis*							
⩾2	14	33	9	31	5	38	0.729

*LDH at diagnosis*							
Greater than ULN	36	88	25	86	11	92	
Extranodal sites >1	10	24	8	28	2	17	0.694

*IPI at diagnosis*							
IPI ⩾3	9	22	5	17	4	33	0.408
Low	17	41	12	43	5	38	
Low intermediate	15	37	11	39	4	31	
High intermediate	7	17	3	11	4	31	
High	2	5	2	7	0	0	
							
*Bulky tumor at diagnosis, cm*							
⩾10	26	70	19	73	7	64	0.699

*Presence of pleural or pericardial effusion at diagnosis*							
Yes	26	60	18	62	8	57	>0.99
							
Rituximab-containing therapy as first-line treatment			33	72	22	54	
Yes	29	66	22	73	7	50	0.177

*Prior RT as first-line treatment*							
Yes	10	23	8	27	2	14	0.462

*First-line treatment*							
R-CHOP	27	63	21	70	6	43	0.107
CHOP	12	28	7	23	5	36	0.475
The second-/third-generation regimens	4	9	2	7	2	14	0.581

*Primary refractory disease*							
Yes	18	41	10	33	8	57	0.191

*Relapse time*							
Relapse <12 months	29	66	19	63	10	71	0.738
Relapse ⩾12 months	15	34	2	22	12	32	

Abbreviations: CHOP, cyclophosphamide, doxorubicin, vincristine and prednisolone; CNS, central nervous system; HDT/ASCT, high-dose chemotherapy followed by autologous stem cell transplantation; IPI, international prognostic index; LDH, lactate dehydrogenase; PS, performance status; R, rituximab; RT, radiotherapy; ULN, upper limit of normal.
