# Supplementary material for: High-dose chemotherapy followed by autologous stem cell transplantation for relapsed/refractory primary mediastinal large B-cell lymphoma
Source: Blood Cancer J. 2015 Dec 4;5(12):e372–. doi: 10.1038/bcj.2015.101 (PMC4735068; doi:10.1038/bcj.2015.101)
Supplement: Supplementary information [file bcj2015101x1.pdf]

Supplemental Method

Treatment and assessment

Patients were treated according to each institution's treatment standards. Clinical data were collected from case report forms. Responses were evaluated by each investigator in accordance with the 1999 International Workshop Criteria[12].

Statistical analysis

In the present analysis, patients with relapsed disease were defined as those with progressive disease after achievement of a partial response (PR) or relapsed disease after achievement of a complete response (CR). Primary refractory disease was defined as patients who did not achieve a CR or PR after first-line treatment. Early relapse was defined as refractory disease or relapse <12 months after diagnosis. Late relapse was defined as relapse ≥12 months after diagnosis. Relapsed or refractory disease was considered to be chemo-sensitive if at least a PR was achieved by salvage chemotherapy before HDT/ASCT; otherwise, the disease was considered chemo-refractory. OS was defined as the period from the day of first relapse or progression of PMBL to the date of death or the last follow-up. PFS was defined as the period from the day of the first relapse or progression of PMBL to disease progression, second relapse, death of any cause, or last date of follow-up. Differences in characteristics between two or more

groups were examined using the chi-square test, the Fisher exact test, or the Mann–Whitney U-test. The cumulative incidence of relapse was estimated with non-relapse death as a competing event. OS and PFS were analyzed using Kaplan-Meier methods, and results were compared using the log-rank test. Univariate Cox regression analyses were performed to assess the effects of prognostic factors. All probability values were two-sided, with a level of 0.05 used as the threshold to indicate statistical significance. Statistical analyses were performed with Stata SE 12 software (StataCorp).

Supplemental Table S1 Risk factors for OS, PFS for patients with relapsed or refractory PMBL treated with HDT/ASCT

	OS			PFS		
	Univariate analysis			Univariate analysis		
Variable	HR	95% CI	P-Value	HR	95% CI	P-Value
Age > 30 years at relapse	0.44	0.10-2.03	0.295	0.5	0.14-1.79	0.288
Male sex	1.23	0.40-3.82	0.717	0.85	0.30-2.40	0.766
Stage III/IV at relapse	0.93	0.28-3.07	0.9	0.8	0.27-2.35	0.687
Presence of pleural or pericardial effusion at diagnosis	1.93	0.58-6.43	0.286	2.69	0.85-8.56	0.093
IPI ≧ 3 at diagnosis	2.15	0.65-7.14	0.213	1.92	0.66-5.64	0.233
Bulky tumor ≥ 10 cm at diagnosis	0.46	0.13-1.63	0.229	0.75	0.23-2.44	0.63
Prior rituximab	0.9	0.29-2.84	0.86	1.24	0.42-3.64	0.697
Prior RT	0.58	0.13-2.63	0.476	0.47	0.11-2.08	0.318
RT after relapse	0.65	0.14-3.00	0.578	1.17	0.37-3.74	0.789
Mediastinum relapse	2.9	0.37-22.52	0.307	3.77	0.50-28.68	0.2
Relapse less than 12 months	5.66	0.73-43.86	0.097	3.53	0.80-15.7	0.097
Refractory to first-line treatment	1.43	0.46-4.44	0.535	1.67	0.60-4.61	0.323

Abbreviations: HDT/ASCT, high-dose chemotherapy followed by autologous stem cell transplantation HR, hazard ratio; IPI, international prognostic index; HDT/ASCT, high-dose chemotherapy followed by autologous stem cell transplantation; CNS, Central nervous system.

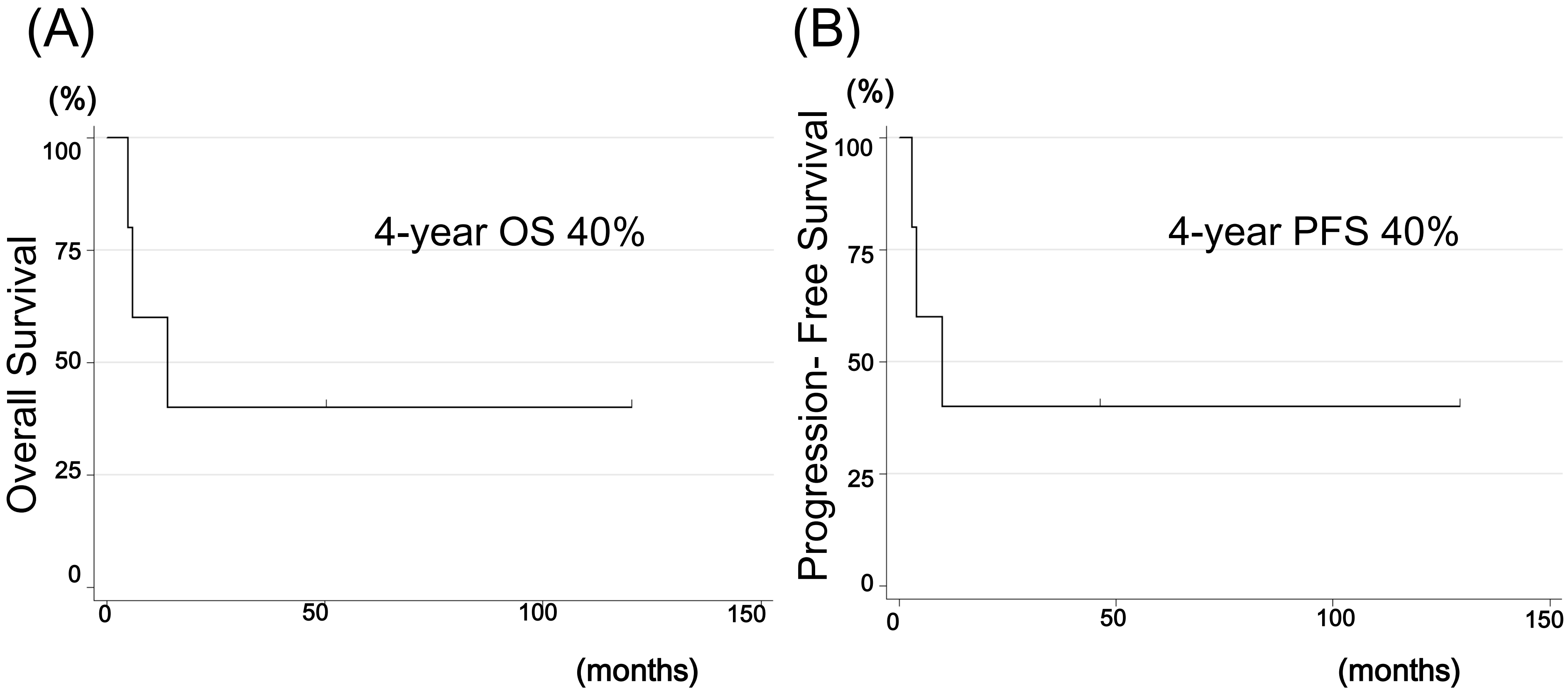

Supplemental Figure S1. Survival after relapse or refractory disease in patients with PMBL treated with allo-HSCT after HDT/ASCT

(A) Overall Survival (N = 5)

(B) Progression-Free Survival (N =5)
